# Autoantibodies to Extracellular Erythrocyte Band 3 Epitopes Are Associated With Anemia in *Plasmodium vivax* Infection

**DOI:** 10.1002/ajh.70463

**Published:** 2026-07-29

**Authors:** Aline Marzano‐Miranda, Vanessa G. Fraga, Cor Jésus F. Fontes, Simone Ladeia‐Andrade, Marcelo U. Ferreira, Daniella C. Bartholomeu, Érika M. Braga

**Affiliations:** ^1^ Department of Parasitology Universidade Federal de Minas Gerais Belo Horizonte Minas Gerais Brazil; ^2^ School of Medical Sciences Federal University of Mato Grosso Cuiabá Mato Grosso Brazil; ^3^ Laboratory of Parasitic Diseases Oswaldo Cruz Institute, Fiocruz Rio de Janeiro Brazil; ^4^ Department of Parasitology Institute of Biomedical Sciences, University of São Paulo São Paulo Brazil

**Keywords:** anemia, autoantibodies, band 3, extracellular loops, *Plasmodium vivax*

## Abstract

*Plasmodium vivax*‐associated anemia is a multifactorial clinical outcome, in which the destruction of uninfected red blood cells (uRBCs) plays a major role in disease development and severity. Here, we identified autoantibody targets within the extracellular loops of Band 3 (B3), a major erythrocyte transmembrane protein. *In silico* epitope prediction identified six of the seven potentially immunogenic extracellular domains of B3. Patients with *P. vivax* infection who were anemic (*n* = 79) and non‐anemic (*n* = 95), and 40 healthy donors from a non‐endemic area were included as negative controls. In addition, 15 anemic and 15 non‐anemic infected individuals were also analyzed immediately before treatment (D0) and on Days 7, 14, and 28 after antimalarial therapy. Synthetic peptides corresponding to the six B3 domains were robustly recognized by autoantibodies from *P. vivax* ‐infected patients. Anemic individuals exhibited prominent autoantibody responses, particularly against loops 4 and 6. IgG3 was the predominant subclass targeting these loops and remained detectable up to 28 days post‐treatment, suggesting a possible role in the persistence of anemia even after parasite clearance. In contrast, IgG1 was the principal subclass recognizing the peptides corresponding to loops 1, 2, and 3, indicating a significant response to senescent domains during *P. vivax* infection. Analysis of IgG subclass against full‐length B3 confirmed the presence of both IgG1 and IgG3 responses. The pro‐inflammatory properties of these IgG subclasses support the hypothesis that anti‐B3 autoantibodies contribute to the immunopathogenesis of *P. vivax* ‐related anemia. Collectively, these findings identify potential targets for future mechanistic investigations and therapeutic intervention strategies.

## 1 Introduction

*Plasmodium vivax* is the most geographically widespread malaria parasite, accounting for approximately 72% of malaria cases in Latin America, with a total of 663 000 cases reported in 2024 [1]. Recent evidence indicates increased severity and mortality associated with *P. vivax* , particularly among children and pregnant women, challenging the perception of this species as relatively benign compared with *Plasmodium falciparum* [2, 3, 4]. Anemia is a multifactorial syndrome and constitutes the most prevalent clinical manifestation of *P. vivax* and *P. falciparum* infections [4, 5, 6, 7]. Among the factors, dyserythropoiesis is an important contributor to anemia during *Plasmodium* infections; it is caused by the direct presence of parasites promoted by the sexual and asexual cycles, as well as by parasite and host products such as hemozoin and cytokines that hamper erythrocyte progenitor development, leading to their apoptosis [8, 9, 10]. Unlike *P. falciparum*, *P. vivax* selectively invades reticulocytes, which constitute only 1%–2% of circulating red blood cells (RBCs); destruction of the infected cells alone cannot account for the extent of hematological impairment observed during infection. Mathematical models estimate erythrocyte removal, especially by splenic macrophages during infections, and for each infected erythrocyte, 17 uninfected ones are destroyed in *P. falciparum* infections, whereas in *P. vivax* this ratio is much higher, at 82:1 [11]. Accumulating evidence suggests that, in addition to multifactorial factors, antibody‐mediated clearance of uninfected red blood cells (uRBCs) is a major contributor to the development of anemia [12, 13, 14, 15, 16]. Antibodies can opsonize uRBCs, promoting their clearance through antibody‐dependent phagocytosis (ADP), antibody‐dependent cellular cytotoxicity (ADCC), and complement activation [17, 18, 19]. Importantly, phosphatidylserine (PS) exposure on the membranes of uRBCs serves as a prominent target for specific antibodies; elevated PS antibody levels are observed during *P. vivax* malaria, facilitating the clearance of these cells [20, 21].

Band 3 (B3) is a major erythrocyte membrane protein, with about 1 000 000 copies per cell [22], and, such as PS, this is a key target of autoantibodies [19, 23]. As a chloride/bicarbonate exchanger, B3 is essential for acid–base homeostasis and CO_2_ transport [24, 25]. This complex molecule comprises a cytoplasmic domain, 14 transmembrane segments, and seven extracellular loops of varying sizes and antigenic properties [26, 27, 28, 29]. Naturally occurring anti‐B3 antibodies are known to mediate the clearance of senescent uRBCs [30, 31, 32, 33] by recognizing the external senescence domain within loop 3, particularly following oxidative glycosylation and protein clustering [34, 35, 36]. Recognition of the internal senescence peptide exposed during erythrocyte aging has also been implicated in this process [37, 38]. However, the role of antibodies targeting other extracellular B3 loops and their potential contribution to the destruction of uRBCs in immunopathological conditions such as *P. vivax* malaria remains largely unclear. Here, we demonstrate that IgG from *P. vivax* malaria patients specifically recognizes extracellular B3 domains on uRBCs. For the first time, we differentiate between potentially pathogenic and physiological IgG responses to B3, revealing sustained and pronounced reactivity to loops 4 and 6 in anemic *P. vivax* patients that persists for at least 28 days following treatment. These responses are predominantly mediated by cytophilic IgG1 and IgG3 antibodies, supporting the involvement of self‐reactive antibodies in uRBC destruction during vivax malaria.

## 2 Methods

### 2.1 Plasma Sample

Blood samples were collected from patients with confirmed *P. vivax* infection by venipuncture into EDTA tubes in two malaria‐endemic regions of Brazil (Table 1). In Area 1 (Hospital Universitário Júlio Muller, Cuiabá, Mato Grosso State), 25 anemic and 37 non‐anemic samples were obtained between February 2006 and January 2008. In Area 2 (Mâncio Lima, Acre State; 07°36′51″ S, 72°53′45″ W), 54 anemic and 58 non‐anemic samples were collected from three urban health centers between June 2014 and July 2015. Additionally, in Area 2, longitudinal samples were collected from 15 anemic and 15 non‐anemic individuals. Each participant underwent clinical assessment, including PCR‐based diagnosis and blood smear analysis. Antimalarial treatment began at baseline (D0) and consisted of 3 days of chloroquine (10 mg/kg) followed by 7 days of primaquine (0.5 mg/kg). Samples were collected at the end of treatment on day seven (D7), with additional samples taken on days 14 (D14) and 28 (D28) after recovery. The treatment protocol was applied consistently to all patients in this study. A complete blood count was performed at baseline (D0) for all patients enrolled in this study [39]. Plasma from 40 healthy donors residing in a non‐endemic region (Belo Horizonte, Minas Gerais state) served as negative controls in serological assays.

**TABLE 1:** Baseline clinical characteristics of the two study populations. Values are presented as median with interquartile range [IQR].

Population parameter	Area 1 Cuiabá, Mato Grosso				Area 2 Mâncio Lima, Acre			
Status	Anemic		Non‐anemic		Anemic		Non‐anemic	
	Woman, Hb ≤ 11	Man, Hb ≤ 12	Woman, Hb > 12	Man, Hb > 14	Woman, Hb ≤ 11	Man, Hb ≤ 12	Woman, Hb > 12	Man, Hb > 14
Sample number	*n* = 7	*n* = 18	*n* = 8	*n* = 29	*n* = 13	*n* = 41	*n* = 28	*n* = 30
Age	39.3 [28–68]	39.3 [18–64]	37.5 [18–48]	39.2 [18–69]	26.4 [8–43]	27.1 [7–60]	27.7 [13–63]	27.7 [9–53]
Hemoglobin level (g/dL)	8.6 [7.1–10.7]	10.7 [8.4–12]	12.9 [12–13.8]	15.6 [14–17.9]	10.4 [9.6–11]	11.1 [7.1–11.9]	12.2 [11.1–13.9]	15 [14.1–17.2]
Hematocrit (%)	26.4 [20.7–33.7]	33 [23.4–40]	38.6 [35.6–45.8]	45.8 [40.7–51.6]	35.5 [29–40.1]	39 [36.2–45]	39.3 [34.4–45.1]	49.6 [42.5–57.9]
Parasitemia (parasites/μL)	14 813 [1400–105 000]	9793 [1115–5535]	1795 [1333–8520]	80 279 [25–10 633]	29 996 [60–100 253]	34 164 [1609–11 914]	26 606 [87–62 232]	27 486 [127–62 162]
Platelets (cells/mm^3^)	176 280 [68 000–321 000]	83 500 [66 000–267 000]	87 153 [49 000–1 580 000]	153 100 [72 000–414 000]	156 234 [87 000–248 000]	171 083 [64 000–307 000]	154 172 [82 000–367 000]	151 913 [90 000–319 000]

### 2.2 In Silico Epitope Prediction and Soluble Peptides Synthesis

The immunogenicity of B3 extracellular domains was evaluated using Bepipred 2.0 for B‐cell epitope prediction, and PyMOL (Schrödinger LLC., Version 2.0) for structural visualization and peptide conformation modeling. Sequences corresponding to the seven extracellular regions (loops 1–7) and the internal senescence peptide were synthesized via solid‐phase peptide synthesis (SPPS), using an automated synthesizer (ResPep SL, Intavis, Germany) on a 10 μmol scale. Briefly, Fmoc groups were removed with 25% 4‐methylpiperidine in dimethylformamide (DMF, Merck), and amino acids were activated using a 1:1 mixture of Oxyma Pure (Merck) and diisopropylcarbodiimide (DIC, Sigma‐Aldrich), then coupled to H‐Rink Amide ChemMatrix resin (Sigma‐Aldrich). Unreacted amino groups were acetylated with 3% acetic anhydride in DMF. Peptides were cleaved from the resin using a cocktail of 92.5% trifluoroacetic acid (TFA), 2.5% water, 2.5% triisopropylsilane (TIPS), and 2.5% thioanisole (Sigma‐Aldrich) with agitation for 3 h. Following precipitation with cold methyl tert‐butyl ether and lyophilization, peptide identity was confirmed by MALDI‐TOF mass spectrometry (Autoflex Speed, Bruker). For analysis, 0.5 μL of concentrated peptide was mixed with 0.25 μL saturated α‐cyano‐4‐hydroxycinnamic acid matrix (Aldrich, Milwaukee, WI) in 50% acetonitrile and 0.1% TFA, then spotted onto an MTP AnchorChip 600/384 plate (Bruker Daltonics) and dried at room temperature.

## 3 Serological Assays

### 3.1 Enzyme‐Linked Immunosorbent Assay for Full‐Length B3 and Extracellular Loops

IgG and IgG subclass responses against purified B3 [40] and its synthetic extracellular loops were assessed to verify the specificity of self‐IgG. As a control, we used p314 peptide, which has been identified as a specific biomarker for *P. vivax* exposure. Briefly, each well of a 96‐well half‐area high‐binding polystyrene microplate (Microlon, Greiner Bio‐One) was coated with 50 ng of B3 at 4°C or 500 ng of synthetic peptides at 37°C in 0.1 M carbonate buffer (pH 9.6) and incubated overnight. After four washes with 0.05% PBST, wells were blocked with 5% (w/v) BSA in PBS (pH 7.4) for 2 h at 37°C. Plasma samples were tested in duplicate at dilutions of 1:100 for total IgG and 1:50 for IgG subclasses in 2.5% PBST/BSA (w/v), followed by incubation for 2 h at 37°C. After serum incubation, four additional washes were performed with 0.05% PBST (Phosphate‐Buffered Saline with Tween‐20). Biotin‐conjugated anti‐human IgG antibodies were diluted in 2.5% PBST/BSA (w/v) at 1:10000 (Sigma‐Aldrich). For the other antibodies, the dilutions were as follows: biotin‐conjugated anti‐human IgG1 at 1:1000; IgG2 at 1:7500; IgG3 at 1:20000; and IgG4 at 1:40000. The diluted antibodies were incubated for 90 min at 37°C, followed by a 1‐h incubation with HRP‐conjugated streptavidin (diluted 1:5000; Sinapse Biotecnologia, Brazil) at 37°C. Finally, the reaction was revealed using the OPD substrate (Sigma‐Aldrich). IgG subclass responses were assessed in 35 of the most reactive anemic *P. vivax* patients from Area 2 and in 12 non‐infected healthy donor samples from Belo Horizonte. For the follow‐up analysis of IgG3 persistence against self‐antigens, we sampled 15 anemic *P. vivax* patients and 12 healthy donors.

### 3.2 Depletion‐ELISA

Serum samples from the 35 most reactive anemic *P. vivax* patients in Area 2 were used to validate the immunodominance of loops 4 and 6 after depletion of IgG antibodies against full‐length B3, either individually or sequentially. In brief, plasma from each test sample was incubated for 1 h at 37°C on plates coated with the extracellular peptide loops 4, 6, or both, thereby capturing antibodies against these epitopes. After this initial incubation, the samples were carefully transferred to fresh plates coated with the same antigens and incubated for an additional hour at 37°C, resulting in a total depletion time of 2 h. Then, samples were gently moved to plates coated with B3 to evaluate the reduction in antibody reactivity. Depletion efficiency was assessed by comparing the IgG reactivity in depleted samples with that of control samples incubated directly on B3‐coated mirror plates without prior depletion on peptide‐coated plates. To examine the impact of double depletion (sequential incubation with loop 4 followed by loop 6), samples were first incubated with each peptide for 1 h. This resulted in a total pre‐incubation time of 4 h (2 h with loop 4 and 2 h with loop 6) before being transferred to B3‐coated plates. Optical density (OD) values were measured at 492 nm using a Multiskan GO Reader (Thermo Scientific) microplate spectrophotometer. The levels of IgG against B3, and the extracellular loop peptides, were expressed as the reactivity index (RI), with the cutoff set at the mean OD of each duplicate sample plus 2 standard deviations, based on sera from 12 malaria‐naïve donors from Belo Horizonte with no history of malaria exposure.

### 3.3 Statistical Analysis

Data analysis was performed using GraphPad Prism 9.0 software (GraphPad Software, CA, USA) and R (version 4.3.0). The Shapiro–Wilk test was used to assess normality. Because the data were non‐normal, group comparisons were conducted using the Kruskal‐Wallis test, followed by Dunn's multiple comparison test. For paired comparisons between two groups, the Wilcoxon test was used. Principal Component Analysis (PCA) was conducted using the factoextra (https://cran.r‐project.org/web/packages/factoextra/index.html), ggplot2 (https://cran.r‐project.org/web/packages/ggplot2/index.html), and MASS (https://cran.r‐project.org/web/packages/MASS/index.html) R packages. A *p*‐value < 0.05 was considered statistically significant.

## 4 Results

### 4.1 IgG Targets the Extracellular B3 Loops

**FIGURE 1:**
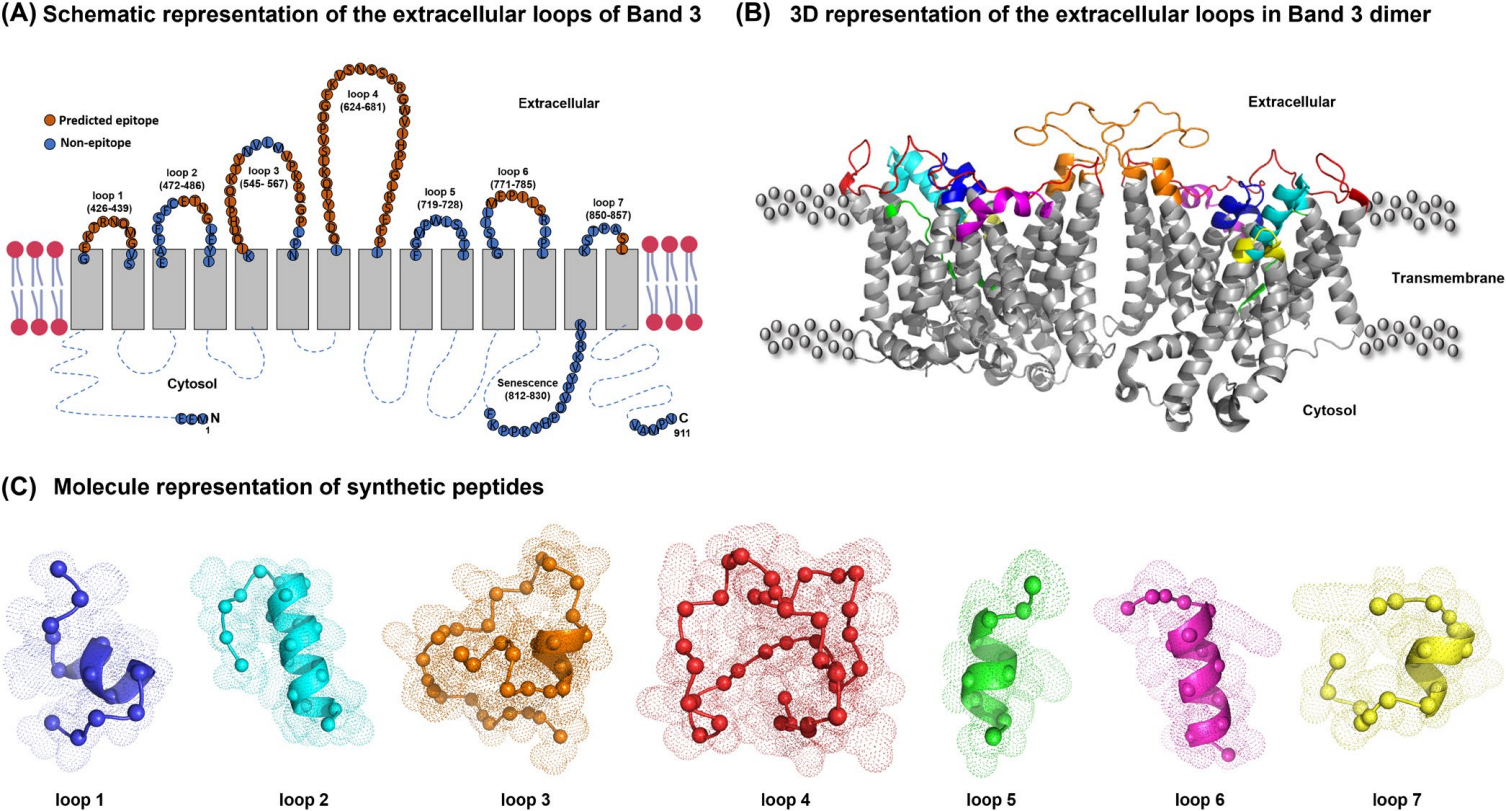
Structural representation and epitope prediction of B and 3 extracellular loops. (A) Schematic illustration of the external loops of the B3 protein, including the amino acid sequences of each synthetic peptide and the predicted B‐cell epitope regions. (B) Three‐dimensional structural model of Band 3 as a dimer embedded in the membrane, illustrating the cytoplasmic, transmembrane, and extracellular domains. The extracellular loops are displayed in their natural orientation on the erythrocyte membrane. (C) Predicted molecular conformations of synthetic peptides corresponding to the extracellular loops are illustrated. Each amino acid is depicted as a sphere within a cartoon model. Dotted shadows represent the spatial position of each atom, illustrating the complete molecular volume. [Color figure can be viewed at wileyonlinelibrary.com]

B‐cell epitope prediction suggested that all extracellular segments of the B3 protein, except loop 5, contain potentially immunogenic epitopes (Figure 1A). These loops are displayed in their natural orientation on the erythrocyte membrane within the three‐dimensional transmembrane B3 dimer structure, which includes cytoplasmic, transmembrane, and extracellular domains (Figure 1B). The predicted conformations of synthetic peptides corresponding to these extracellular sequences were also presented (Figure 1C).

**FIGURE 2:**
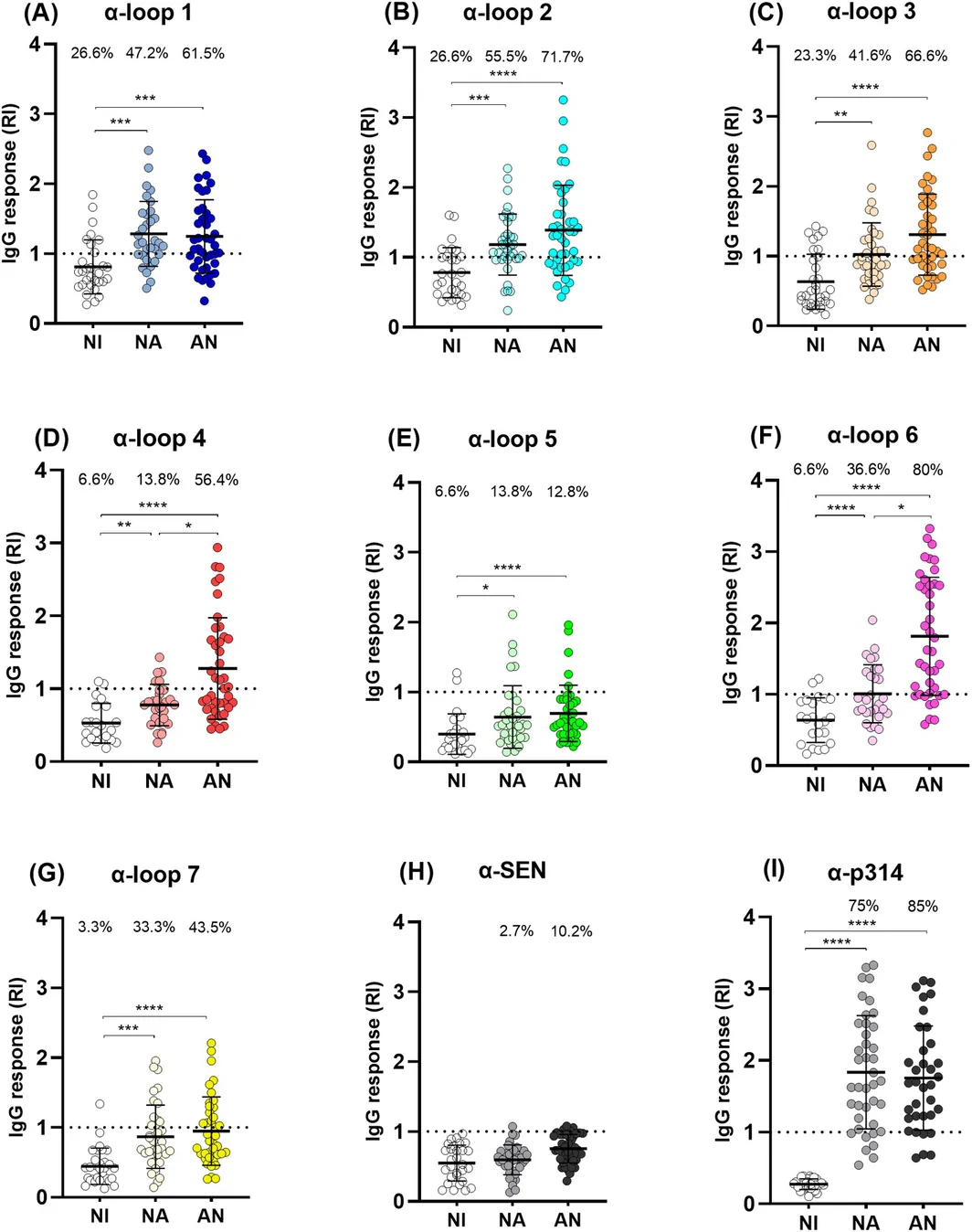
IgG responses to extracellular loops of B3 and a senescence‐associated internal peptide. (A–H) Serological responses to individual synthetic peptides that correspond to the extracellular loops of B3 and one internal peptide related to erythrocyte senescence were evaluated in plasma samples from three groups: non‐infected individuals (NI = 30), non‐anemic patients infected with *P. vivax* (NA = 37), and anemic patients infected with *P. vivax* (AN = 25) from Area 1. (I) To evaluate parasite‐specific IgG responses, we assessed reactivity to p314, a peptide derived from the merozoite surface protein 1 (*Pv*MSP‐1) of *P. vivax* . Antibody levels are expressed as a Reactivity Index (RI). Cut‐off values were established based on the mean plus two standard deviations of the RI values from 10 healthy donors. We calculated the percentage of positive responses as the proportion of samples exceeding the cut‐off compared to the total number of samples in each group. Statistical comparisons among the three groups were conducted using the Kruskal‐Wallis test followed by Dunn's post hoc test. Significance levels were set at *p < 0.01, ***p < 0.001, and ****p < 0.0001. [Color figure can be viewed at wileyonlinelibrary.com]

Samples from Area 1 (Table 1) were used to screen for IgG reactivity against the external domains of B3. Although autoantibodies against the extracellular regions of B3 were physiologically detected in some healthy donors, *P. vivax* infection significantly increased IgG detection across the seven external domains analyzed (Figure 2A–G). Anemic patients exhibited particularly high levels of autoantibodies against loops 4 and 6, which were the most recognized among all groups. In comparison with healthy donors, IgG reactivity against loops 4 and 6 was significantly elevated in anemic patients (*p* < 0.0001 for both). Furthermore, anemic *P. vivax* patients displayed higher autoantibody levels against loop 4 (*p* = 0.0204) and loop 6 (*p* = 0.0236) than non‐anemic malaria patients (Figure 2D,F). An internal senescence peptide from B3 was used as a physiological control; no significant differences were observed between groups, and all three experimental groups remained below the cut‐off threshold (Figure 2H). As a *P. vivax* ‐specific serological control, we used the peptide p314 derived from the *Pv*MSP‐1 surface protein. The results revealed no significant difference in the specific IgG responses to the p314 peptide between anemic and non‐anemic patients (Figure 2I). However, both patient groups demonstrated significantly higher responses than non‐infected individuals (*p* < 0.0001), as previously illustrated by our group.

The IgG response to the relevant external loops was also confirmed for samples from patients in Area 2 (Figure S1A–C). Anemic *P. vivax* patients exhibited significantly higher IgG levels against external loops 4 and 6 (*p* < 0.0001) compared to non‐infected individuals. Non‐anemic malaria patients showed lower self‐IgG responses against loops 4 (*p* = 0.0009) and 6 (*p* = 0.0049) than anemic patients. PCA analysis confirmed the significance of these loops in the IgG responses of *P. vivax* anemic patients. Based on the PCA analysis, IgG responses to the B3 external loops were clustered according to the clinical anemia status (Figure S1D).

### 4.2 Dual Response Against B3 Extracellular Domains: Possible Threshold Between Senescence and Pathogenesis

**FIGURE 3:**
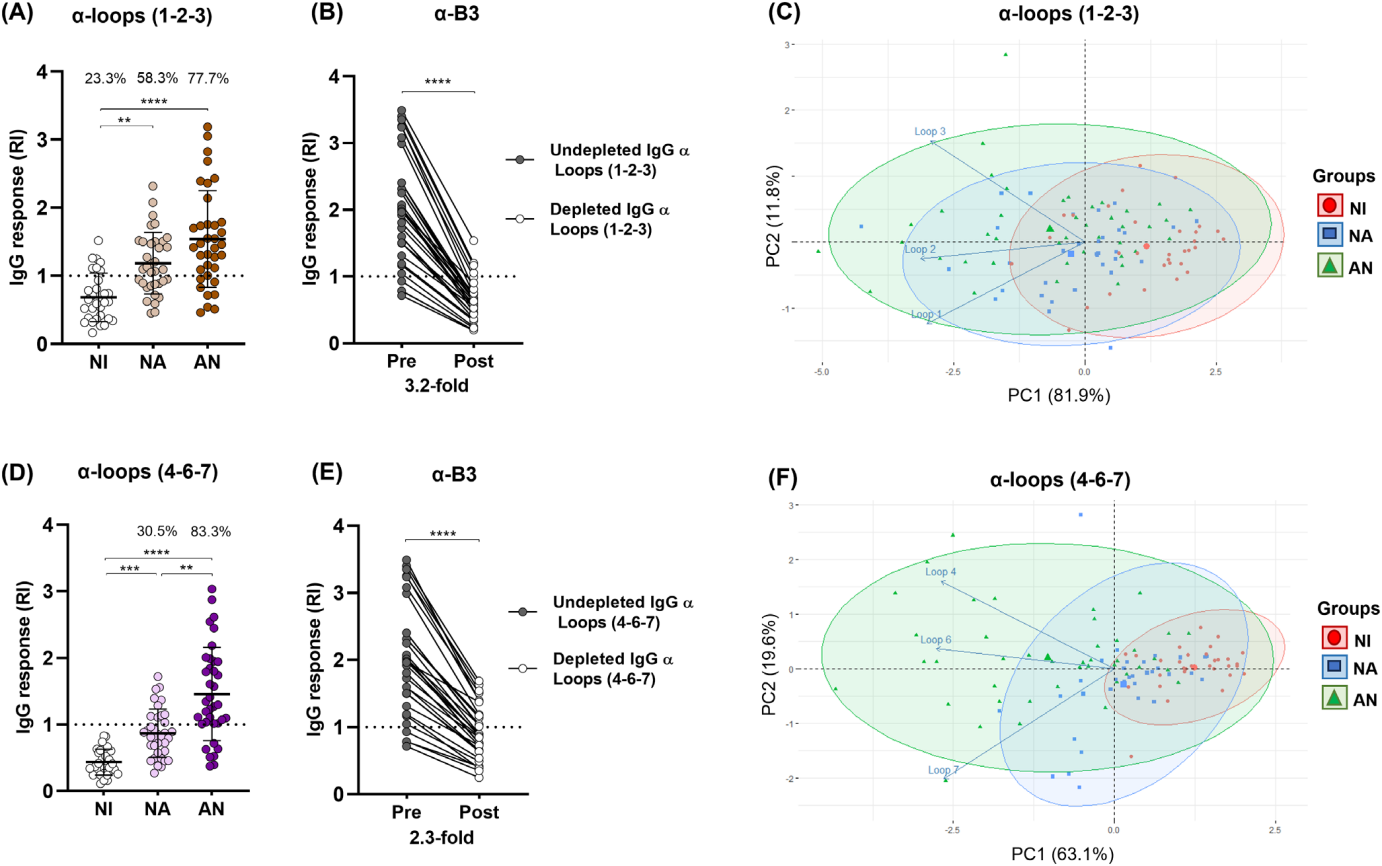
IgG responses to pooled extracellular B3 loops and their contribution to the Overall anti‐nRBC reactivity. (A, D) IgG responses to peptide Pools 1 and 2 were evaluated in plasma samples from healthy donors (NI = 30), non‐anemic (NA = 37), and anemic (AN = 25) *P. vivax* ‐infected patients. Pooled loops (1‐2‐3) comprise the combination of peptides corresponding to loops 1, 2, and 3; Pooled loops (4‐6‐7) comprise the combination of peptides corresponding to loops 4, 6, and 7. Antibody levels are expressed as Reactivity Index (RI). Statistical comparisons were made using the Kruskal–Wallis test followed by Dunn's post hoc test. (B, E) Depletion ELISA was performed with anemic *P. vivax* samples (*n* = 35) to assess the contribution of antibodies targeting Pooled loops (1‐2‐3) and Pooled loops (4‐6‐7) to the total anti‐nRBC IgG response. The Wilcoxon test was used for pairwise comparisons. (C, F) Principal Component Analysis (PCA) was conducted using ELISA‐derived serological data from Area 1 samples to evaluate the distribution and clustering of IgG responses to Pooled loops (1‐2‐3) and Pooled loops (4‐6‐7). *p < 0.01; ***p < 0.001; ***p < 0.0001. [Color figure can be viewed at wileyonlinelibrary.com]

Analyzing pooled antigens based on individual peptide serological response patterns reveals that antibody reactivity against loops 1, 2, and 3 may indicate a pre‐existing physiological response found in 23% of healthy donors, which becomes significantly enhanced during infection (*p* = 0.003), particularly in anemic patients (*p* < 0.0001) (Figure 3A). In contrast, the same analytical approach identified loops 4, 6, and 7 as unrelated to physiological status but directly associated with anemia, potentially triggered by *P. vivax* infection (*p* < 0.0001) (Figure 3D). Furthermore, depletion ELISA using the overall IgG response to B3 confirmed a significant reduction in IgG responses, 3.2‐fold and 2.3‐fold, respectively, against the external regions represented by pooled loops 1–2–3 (*p* < 0.0001) and pooled loops 4–6–7 (*p* < 0.0001) (Figures 3B,E). Indeed, PCA analysis confirmed that autoantibodies from anemic patients preferentially recognize the B3 external pooled loops 4–6–7, rather than those represented by pooled loops 1–2–3 (Figures 3C,F).

### 4.3 Immunodominance of Extracellular Loops 4 and 6

**FIGURE 4:**
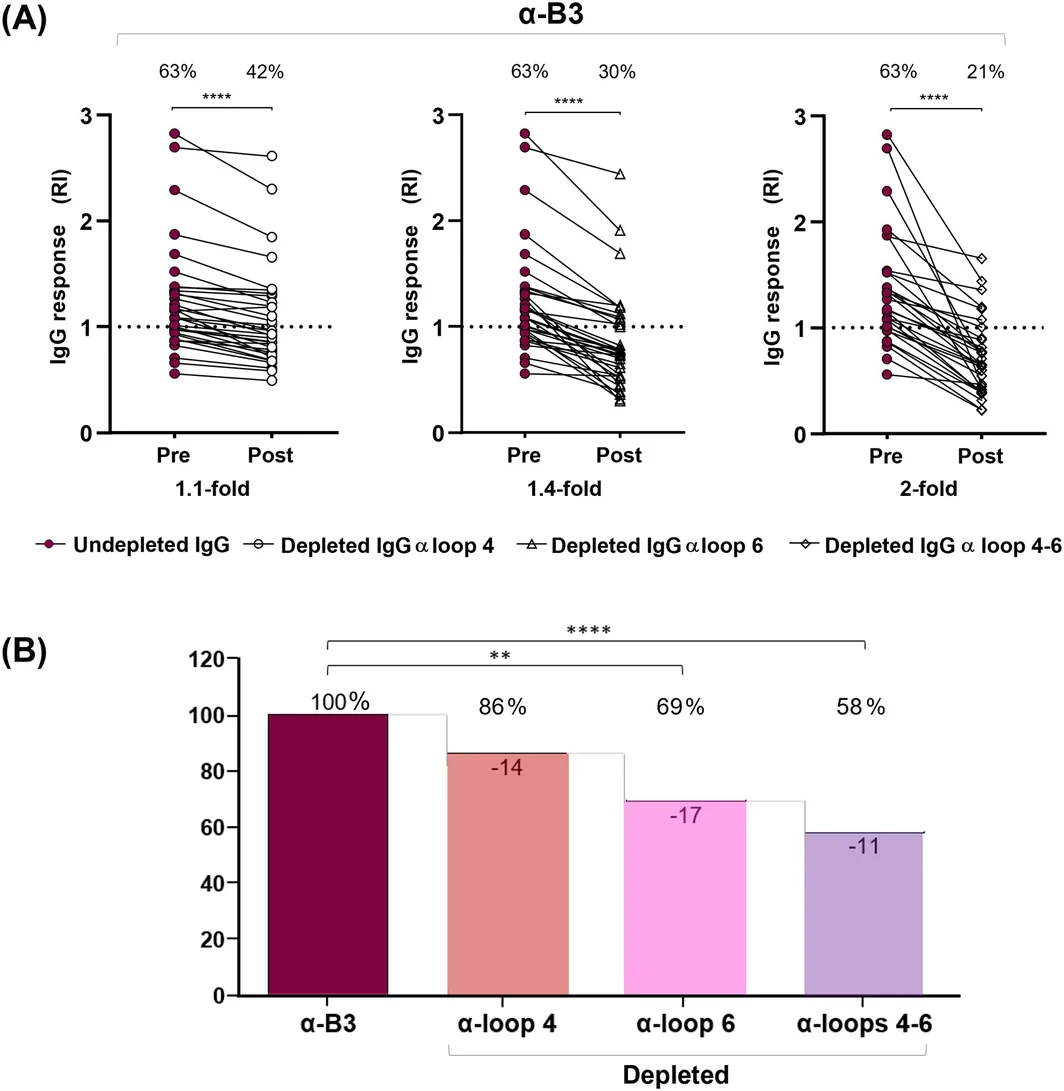
Analysis of IgG responses to Band 3 loops 4 and 6 in anemic *P. vivax* Patients. (A) Depletion ELISA was performed to evaluate the contribution of IgG antibodies specific to loops 4 and 6 to the overall response against the full‐length Band 3 protein. Paired plasma samples from anemic *P. vivax* ‐infected patients (*n* = 35) were analyzed before and after antibody depletion. Individual reductions in reactivity following depletion with loop 4, loop 6, and both peptides combined are shown. (B) The median reactivity values were converted into percentages to illustrate the relative decrease in IgG binding after depletion. Statistical analysis was performed on IgG absorbance values to confirm the significance of the reduction in reactivity. Comparisons were made using the Wilcoxon test for paired statistical analysis and the Kruskal‐Wallis test, followed by Dunn's post hoc test for comparisons among three groups. *p < 0.01; ***p < 0.001; ****p < 0.0001. [Color figure can be viewed at wileyonlinelibrary.com]

To evaluate the significance of IgG targeting loops 4 and 6 relative to the full‐length B3 protein, we performed a depletion ELISA. Pre‐incubation with either loop 4 or loop 6 peptides resulted in a significant reduction in B3 reactivity among anemic *P. vivax* patients, with decreases of 1.1‐fold and 1.4‐fold, respectively (*p* < 0.0001), indicating effective capture of specific IgG. When both peptides were used together for depletion, the reduction in reactivity was even more pronounced across all individual samples, achieving a 2‐fold decrease (*p* < 0.0001) (Figure 4A). To assess the impact of IgG depletion on B3, we measured the decrease in reactivity after incubation with individual peptides or with both loops combined. The baseline for comparison was the absorbance (OD) of B3 IgG reactivity without pre‐incubation, measured at 0.71 (considered 100%). Pre‐incubation with loop 4 resulted in a 14% reduction in reactivity (median OD: 0.61), while loop 6 caused a 31% decrease (median OD: 0.50). When both loops 4 and 6 were pre‐incubated sequentially, the median reactivity was reduced by 42% (median OD: 0.43) (Figure 4B). A significant decrease in IgG reactivity was observed after incubation with loop 6 (*p* = 0.0034), and an even greater reduction was noted after incubation with combined loops (*p* < 0.0001) (Figure 4B).

### 4.4 B3‐IgGs Persist in Anemic Patients After *P. vivax* Treatment

**FIGURE 5:**
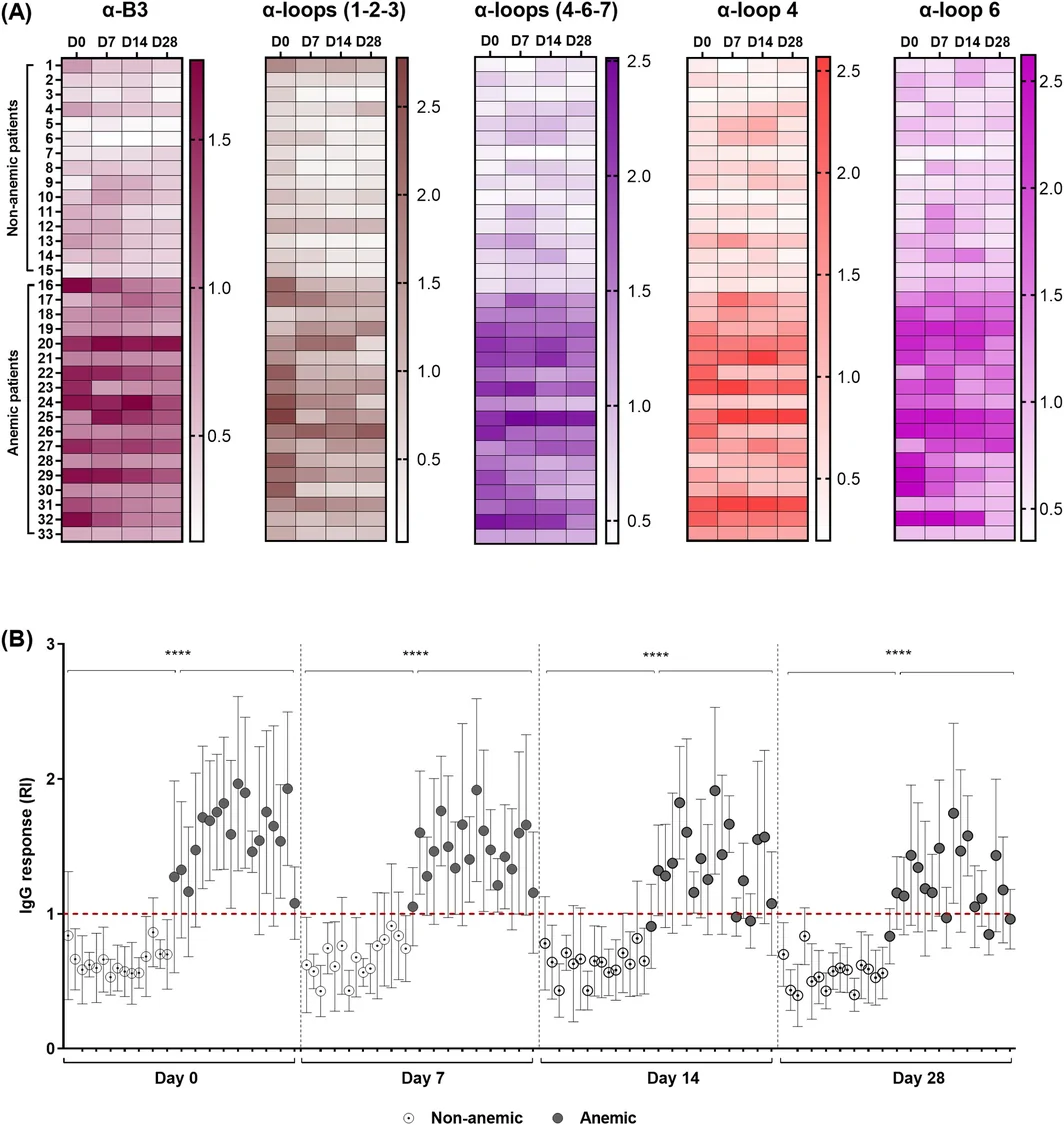
Longitudinal analysis of self‐reactive IgG persistence following *P. vivax* treatment. (A) Heatmap representation of IgG reactivity at baseline (Day 0, D0), and 7, 14, and 28 days post‐treatment (D7, D14, D28) in 15 non‐anemic and 15 anemic *P. vivax* ‐infected patients. Antibody reactivity was assessed against full‐length B3 protein, the individual peptides loop 4 and loop 6, and against the pooled loops (1‐2‐3) and pooled loops (4‐6‐7). (B) Overlapping IgG positivity across all antigenic targets is shown for individual patients over the follow‐up period (D0, D7, D14, D28), demonstrating the relative persistence of self‐reactive antibodies after parasite clearance in anemic *P. vivax* patients compared with non‐anemics. Reactivity Index (RI) values were calculated using a cut‐off determined from the IgG responses of 10 healthy donors. Grouped comparisons were made using the Two‐way ANOVA. Samples were considered positive when RI > 1.*p < 0.01; ***p < 0.001; ****p < 0.0001. [Color figure can be viewed at wileyonlinelibrary.com]

Follow‐up samples were collected during the acute infection (Day 0, D0) and at 7, 14, and 28 days post‐treatment (D7, D14, D28) from both anemic and non‐anemic *P. vivax* patients from Area 2 (Table 1). These samples were assessed for IgG reactivity to full‐length B3, individual loops 4 and 6, and combined loops (1–2–3) and (4–6–7). The findings indicated that patients with anemia exhibited elevated IgG responses to self‐antigens for up to 28 days following infection, even after receiving specific treatment for *P. vivax* (Figure 5A). Statistical analysis of all targets revealed that self‐directed IgG responses were significantly higher in anemic individuals than in their non‐anemic counterparts throughout the follow‐up period (*p* < 0.0001). Furthermore, anemic patients displayed a significant humoral immune response, with most samples exceeding the cut‐off levels for all tested antigens (Figure 5B).

### 4.5 Cytophilic IgG Subclasses Bind to the B3 Extracellular Loops in Anemic *P. vivax* Patients

**FIGURE 6:**
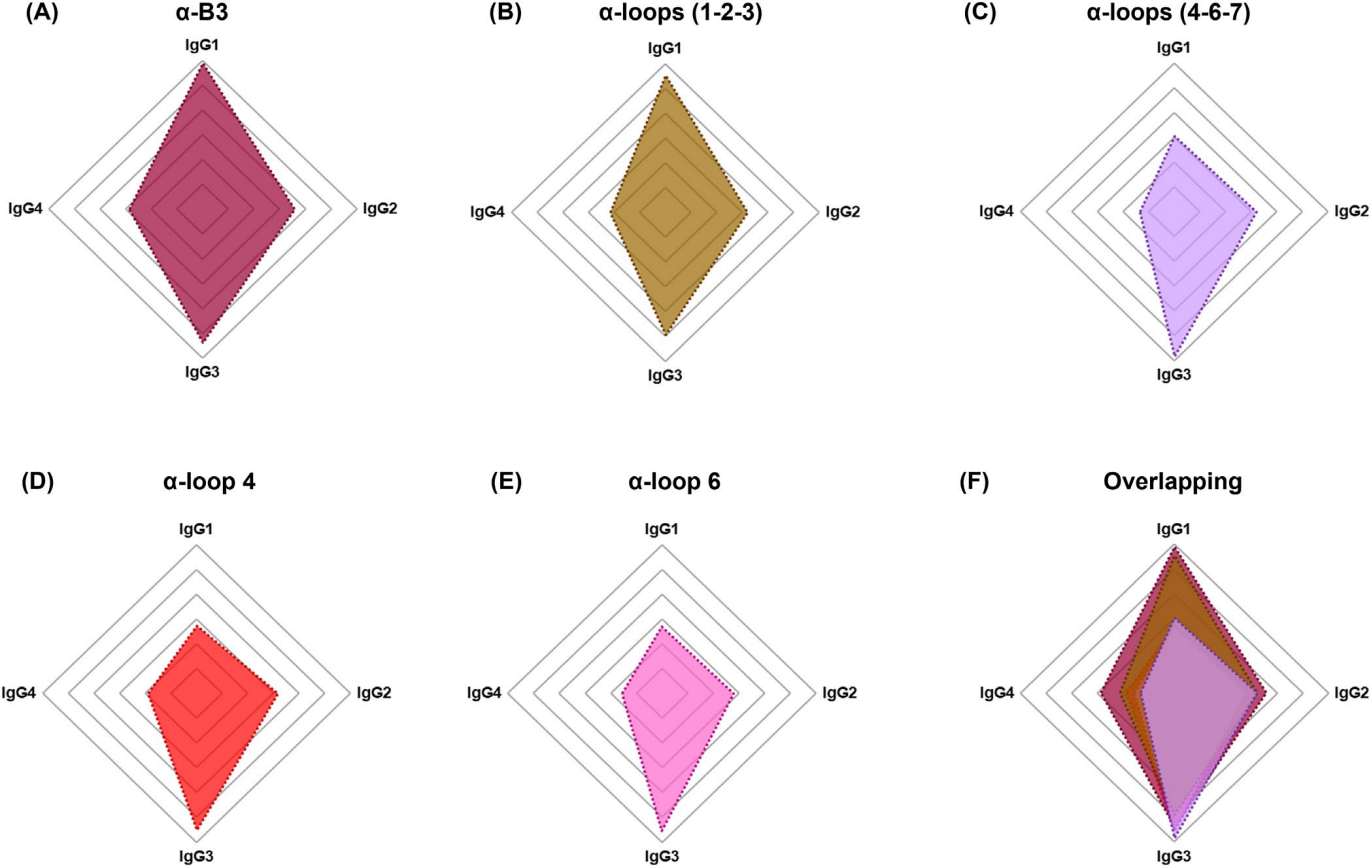
IgG subclass distribution against B3 and extracellular loops. Radar plots illustrate the distribution frequencies of IgG subclasses (IgG1, IgG2, IgG3, and IgG4) directed against full‐length Band 3, pooled loops (1‐2‐3); pooled loops (4‐6‐7); loop 4, and loop 6 in plasma samples from anemic *P. vivax* patients (*n* = 35). Panels (A–E) depict the subclass profiles for each antigen at baseline. Panel (F) presents the merged reactivity for all evaluated targets. Each radar chart is divided into concentric rings representing 20% increments in response magnitude, with axis values increasing by 0.2 per interval. [Color figure can be viewed at wileyonlinelibrary.com]

The IgG1 and IgG3 subclasses are the most prominent in recognizing the full‐length B3 protein in the plasma of anemic individuals infected with *P. vivax* . In contrast, IgG2 and IgG4 are present at lower levels (Figure 6A). In examining the combined antigens, a specific pattern emerges: IgG1 is predominant against pooled loops (1–2–3), followed by IgG3. In comparison, the response to pooled loops (4–6–7) is primarily driven by IgG3 (Figure 6B,C). Interestingly, the analysis of IgG responses in anemic patients to the extracellular loops 4 and 6 of B3 shows a pronounced IgG3 response (Figure 6D,E). Anemic patients exhibit significantly higher levels of IgG1 and IgG3 autoantibodies compared to healthy donors (Figure 6F). Since IgG3 typically declines more rapidly in circulation, we assessed post‐infection IgG3 levels targeting B3, loops 4 and 6, and the pooled loops (1–2–3) and (4–6–7). IgG3 levels remained above the cut‐off for at least 28 days after infection in samples from anemic patients for all antigens (Figure S2). These findings suggest that IgG1 and IgG3 may contribute to the self‐reactive antibody response associated with anemia during both the acute phase and the post‐infection period in *P. vivax* infections.

## 5 Discussion

*Plasmodium vivax*‐associated anemia cannot be explained solely by direct parasite‐induced hemolysis but also involves immune‐mediated mechanisms targeting uRBCs. Our findings suggest a significant autoantibodies response against the erythrocyte membrane protein B3, particularly directed toward extracellular loops 4 and 6 in anemic patients. These autoantibodies, predominantly of the pro‐inflammatory IgG1 and IgG3 subclasses, persist beyond parasite clearance, suggesting their potential role in opsonizing uRBCs and promoting their clearance through erythrophagocytosis, thereby contributing to anemia development and persistence.

The natural production of B3 autoantibodies is a physiological process in which low concentrations of these antibodies bind to oxidized extracellular B3 domains, promoting the clearance of senescent erythrocytes by splenic macrophages [41, 42]. During *P. vivax* infection, autoantibody levels are markedly elevated, particularly among individuals presenting with anemia and thrombocytopenia [14, 43, 44]. This increase may result from parasite‐induced systemic inflammation [45, 46, 47], which can disrupt immune tolerance by enhancing both the presentation of self‐erythrocyte antigens and polyclonal B‐cell activation [48]. Additionally, the phagocytic checkpoint response involved in clearance of debris following the rupture of infected erythrocytes may facilitate the presentation of self‐antigens together with parasite‐derived components, thereby further promoting RBC‐reactive IgG production [49, 50]. Notably, the presence, upregulation, and pathogenic potential of these autoantibodies have been documented not only in malaria [20, 21, 51] but also in autoimmune hemolytic disorders [52, 53, 54].

To elucidate B3 epitopes implicated in erythrocyte opsonization, we performed in silico B‐cell epitope prediction, identifying putative epitopes within six of the seven extracellular regions of B3 as potential autoantibody targets. Using synthetic peptides corresponding to these external domains, we observed that anemic patients exhibited markedly elevated autoantibody responses, with seropositivity exceeding 50% for five of the six extracellular regions evaluated. Conversely, a synthetic peptide representing an internal senescence domain of B3, which becomes exposed to the erythrocyte surface during cellular aging [38], did not display significant reactivity differences according to anemia status. Likewise, a peptide derived from *Pv*MSP1 (peptide p314), a recognized marker of *P. vivax* exposure [55], elicited comparable humoral responses in both anemic and non‐anemic cohorts. Taken together, these findings suggest that the observed variation in autoantibody reactivity is more closely associated with anemia status than with the overall magnitude of the anti‐parasite antibody response.

Analysis of IgG responses to the B3 loops revealed a consistent pattern. Both non‐anemic and anemic patients infected with *P. vivax* exhibited IgG responses to loops 1, 2, and 3, with anemic patients showing higher reactivity. Interestingly, even malaria‐naive individuals also demonstrated detectable IgG reactivity to these loops, albeit at lower levels. PCA revealed substantial overlap among IgG profiles across all groups, suggesting the existence of a shared immunoreactivity pattern. Because B3 is associated with erythrocyte senescence checkpoint mechanisms [25, 41, 42], this antibody response may represent a physiological autoreactive response that becomes more amplified during *P. vivax* infection due to systemic inflammation [56, 57, 58]. The inflammatory environment induced by *P. vivax* infection may promote the expansion of pre‐existing B‐cell clones that naturally produce basal levels of self‐antibodies, resulting in their differentiation into antibody‐secreting plasmablasts [59, 60]. Supporting this hypothesis, IgG1 has been identified as the predominant subclass recognizing loops 1–3. IgG1 is characterized by a long serum half‐life and is associated with memory B cell responses following previous antigen exposure [61, 62]. Although IgG1 responses may be protective or physiologically beneficial under homeostatic conditions [63], elevated levels directed against self‐antigens during *P. vivax* infection may contribute to pathogenic processes, particularly given the cytophilic and pro‐inflammatory properties of this subclass and its ability to mediate antibody‐dependent effector functions [64, 65].

Among the B3 extracellular regions, the heightened IgG reactivity to loops 4 and 6 observed in anemic *P. vivax* patients suggests a potential association between these specific epitopes and malaria‐related anemia. These regions have been described as potential interaction sites between *P. vivax* and reticulocytes during invasion [66, 67, 68]. Their likely involvement in host–parasite interactions may increase their immunological visibility under inflammatory conditions, favoring the production of autoantibodies directed against uRBCs. The potential role of antibodies targeting loops 4 and 6 is further supported by the identification of IgG3 as the predominant subclass binding these regions. IgG3 is often among the earliest IgG subclasses produced during inflammatory immune responses and is often associated with acute‐phase immunity [69, 70]. Similar to IgG1, IgG3 exhibits cytophilic properties and is crucial for pro‐inflammatory antibody‐dependent effector functions, such as phagocyte activation [71, 72]. Therefore, the predominance of IgG3 suggests an ongoing or recently vigorous immune response, which may facilitate the immunopathological elimination of uRBCs in anemic patients with *P. vivax* infection.

Malaria‐associated anemia often persists after parasite clearance [17, 73], highlighting the critical contribution of immune‐mediated mechanisms, particularly antibody response, to this pathological process. Here, we assessed the B3 IgG response using follow‐up samples from both anemic and non‐anemic patients infected with *P. vivax* . Autoantibodies targeting the native B3 and its extracellular loops remained above the established cut‐off for at least 28 days following anti‐malarial treatment in a subset of patients who were anemic at the time of diagnosis. These findings have significant implications, as previous studies in both *P. vivax‐*infected individuals and murine malaria models have demonstrated that reduced hemoglobin and hematocrit levels can persist and may even worsen after complete parasite clearance [74, 75]. Our results demonstrate that autoreactive IgG directed against B3 is increased in *P. vivax* –anemic patients, predominantly belong to cytophilic subclasses IgG1 and IgG3, and persists beyond parasite clearance.

Taken together, these features support the hypothesis that self‐reactive antibodies targeting erythrocyte surface antigens can contribute to the clearance of uRBCs during both the acute and post‐infection phases of *P. vivax* malaria. However, some limitations should be acknowledged. First, this study focuses exclusively on *P. vivax* , the most prevalent malaria‐causing species in Brazil. Second, the limited number of follow‐up samples and potential variability in antibody levels across different transmission settings underscore the need for further research. Finally, future assays are essential to establish the pathogenic role of antibodies against Band 3 during *P. vivax* infections.

## 6 Conclusion

This study provides novel insights into the mechanisms underlying *P. vivax* ‐associated anemia by elucidating the role of autoantibodies targeting the extracellular domains of B3, a major erythrocyte transmembrane protein. Using synthetic peptides representing B3 extracellular loops, we mapped specific epitopes recognized by self‐reactive IgG, primarily of the cytophilic IgG1 and IgG3 subclasses, which are associated with pro‐inflammatory activity and Fc receptor‐mediated phagocytosis. Notably, these autoantibody responses persisted for at least 28 days after parasite clearance, supporting their potential contribution to anemia beyond the acute infection. Taken together, our findings establish B3 extracellular epitopes as targets of autoreactive antibodies in vivax malaria, provide new tools for investigating malaria‐associated autoimmunity, and highlight a potential mechanism contributing to the persistence of anemia following parasite clearance.

## Funding

This work was supported by the Fundação de Amparo à Pesquisa do Estado de Minas Gerais—FAPEMIG (grant number APQ03740‐23), and Conselho Nacional de Desenvolvimento Científico e Tecnológico—CNPq (grant number 402696/2024‐7). Fieldwork in Acre was funded by the Ministry of Health of Brazil and the Conselho Nacional de Desenvolvimento Científico e Tecnológico‐CNPq, Brazil (grant number 404067/2012‐3). We acknowledge senior researcher scholarships from CNPq for D.C.B., M.U.F., and E.M.B.

## Ethics Statement

This study received ethical approval from the Ethics Committee of the National Information System on Research Ethics Involving Human Beings (SISNEP‐CAAE 01496013.8.0000.5149 and 69999423.6.0000.5149). Written informed consent was obtained from all participants before blood collection. Individuals displaying severe malnutrition or co‐infection with HIV or hepatitis viruses were excluded from participation.

## Conflicts of Interest

The authors declare no conflicts of interest.

## Supporting information


**Figure S1:** IgG serological responses to critical external Band 3 targets in *P. vivax* ‐infected patients from area 2.

**Figure S2:** IgG3 reactivity across the follow‐up period.

## Data Availability

All data generated or analyzed during this study are included in this published article (and its Supporting Information files).
